# The cryoprotectant system of Cope’s gray treefrog, *Dryophytes chrysoscelis*: responses to cold acclimation, freezing, and thawing

**DOI:** 10.1007/s00360-018-1153-6

**Published:** 2018-03-17

**Authors:** M. Clara F. do Amaral, James Frisbie, David L. Goldstein, Carissa M. Krane

**Affiliations:** 1 Department of Biology, Mount St. Joseph University, 5701 Delhi Ave, Cincinnati, OH 45233 USA; 2 Department of Biology, University of Dayton, 300 College Park Ave., Dayton, OH 45469 USA; 3 Department of Biological Sciences, Wright State University, 3640 Colonel Glenn Hwy, Dayton, OH 45435 USA

**Keywords:** *Dryophytes chrysoscelis*, Freeze tolerance, Cryoprotectant, Glycerol, Glucose, Urea.

## Abstract

Cope’s gray treefrog (*Dryophytes chrysoscelis*) is one of few freeze-tolerant frogs that mobilize glycerol as a cryoprotectant, yet cold and freezing-induced accumulation of this and other osmolytes has received little attention in this species. This study investigated the development of freeze tolerance in *D. chrysoscelis*, analyzing the response of the cryoprotectant system to cold acclimation, freezing, and thawing. Glycerol production was low and unresponsive to acclimation temperature, or duration of acclimation to 5 °C, except for one cold-acclimated frog that presented elevated glycerol in plasma, liver, and skeletal muscle. Curiously, glycerol concentration in skeletal muscle was higher than that of plasma and liver, in both warm- and cold-acclimated frogs, suggesting glycerol synthesis in muscle. Urea concentration in plasma doubled in response to cold acclimation but did not change during freezing. Freezing induced hepatic glycogen catabolism and an increase in glycerol and glucose in several tissues, although the mobilization dynamics differed between these cryoprotectants, possibly as a result of different transport mechanisms. Although hepatic glucose mobilization was of considerable magnitude, glucose accumulation in peripheral tissues was low and was surpassed by that of glycerol and urea. The muscle production of glycerol and the cold-induced accumulation of urea imply a role for skeletal muscle metabolism in the mobilization of cryoprotective solutes in *D. chrysoscelis*. The cryoprotectant system of *D. chrysoscelis* is complex, highly variable, and unique, with glycerol, glucose, and likely urea serving as cryoprotectants.

## Introduction

Among temperate vertebrates, a small number of anurans survive the low temperatures of winter by tolerating freezing of up to 65% of their body fluids, despite the several physical, biochemical, and molecular challenges associated with internal ice formation (Costanzo and Lee 2013; Storey and Storey 2017). One of the strategies that contributes to freezing survival is the accumulation of cryoprotectants: low molecular weight compounds that colligatively reduce ice formation, may serve as energetic substrates, and possibly stabilize proteins and membranes (Costanzo and Lee 2013; Storey and Storey 2017).

The ability to survive somatic freezing has a strong seasonal component (Layne and Lee 1989; Costanzo et al. 2014), with winter-conditioned frogs showing enhanced freeze tolerance compared with summer animals (Layne and Lee 1989). The progressive decrease in temperature that precedes winter months influences the accumulation of energetic substrates (Koskela and Pasanen 1975; Scapin and Di Giuseppe 1994; do Amaral et al. 2016), and in freeze-tolerant frogs, primes the cryoprotectant system and the associated enzymatic machinery for survival to subzero temperatures (Swanson et al. 1996; Costanzo et al. 2014; do Amaral et al. 2016).

In freeze-tolerant anurans, accumulation of cryoprotective solutes may occur during fall, as in the case of urea accumulation by the wood frog, *Rana sylvatica* (Costanzo and Lee 2005), and glycerol accumulation by the gray treefrog complex (Layne 1999; Irwin and Lee 2003), which includes *Dryophytes chrysoscelis* and *D*. v*ersicolor*, formerly *Hyla chrysoscelis* and *H. versicolor* (Duellman et al. 2016). At the onset of freezing, hepatic glycogen is catabolized to generate glucose in several freeze-tolerant frogs (Storey and Storey 2017), and possibly glycerol in *D. versicolor* (Layne 1999). In contrast, it is not clear if muscle directly synthesizes cryoprotectants (Storey and Storey 2004), although muscle protein catabolism likely supplies the substrate necessary for urea synthesis in winter-conditioned *R. sylvatica* (Costanzo et al. 2013).

The stimulus and dynamics of cryoprotectant accumulation and clearance are not well understood in *D. chrysoscelis*. Previous studies present conflicting reports regarding the anticipatory accumulation of glycerol in this species (Costanzo et al. 1992; Irwin and Lee 2003; Zimmerman et al. 2007), possibly as the result of distinct acclimation regimes preceding freezing (Layne and Jones 2001), although additional factors are likely involved (Layne and Stapleton 2008). Moreover, heretofore freezing-induced accumulation of glycerol has not been detected in *D. chrysoscelis* (Costanzo et al. 1992; Irwin and Lee 2003), although it is known to occur in *D. versicolor* (Storey and Storey 1985; Layne 1999; Layne and Stapleton 2008). Curiously, cryoprotectant clearance during thawing has received little attention in either species.

This study investigated how acclimation, freezing, and thawing affected the cryoprotectant system in *Dryophytes chrysoscelis*. It was hypothesized that cold acclimation would induce accumulation of glycerol and the anticipatory accumulation of glucose and urea was also investigated. In addition, it was hypothesized that freezing would induce cryoprotectant mobilization from hepatic sources, followed by clearance of these compounds during thawing.

## Materials and methods

### Experimental animals

*Dryophytes chrysoscelis* were collected, under a permit from the Ohio Division of Wildlife, from ponds in Greene County, Ohio, during the summer months. Animals were transported to the laboratory at Wright State University, where they were housed in plastic cages with free access to water. Frog rearing and experiments involving live animals were conducted at Wright State University with the approval of the Laboratory Animal Care and Use Committee (LACUC).

### Acclimation experiment

*Dryophytes chrysoscelis* were acclimated to different temperatures as previously described (Goldstein et al. 2010). Initially, frogs were warm-acclimated to 22 °C, under a 12:12 h light–dark regime, and fed crickets thrice weekly. A group of frogs (*N* = 6) was sampled after a minimum of 12 weeks under warm acclimation (herein called “warm frogs”). In late October, a random subset of animals was transferred to a refrigerated room and was cold-acclimated by being progressively cooled to 5 °C: during a 2-month period the environmental temperature changed from 22, to 20, to 15, to 8 °C, decreasing every 2 weeks, and finally remained at 5 °C for 2 weeks. Cold acclimation was accompanied by a shift to an 8:16 h light–dark regime. Frogs held at 5 °C were not kept in constant darkness (as in other studies, i.e., Costanzo et al. 2013 and; do Amaral et al. 2016), to replicate acclimation regimes from our laboratory (Zimmerman et al. 2007; Goldstein et al. 2010), and while photoperiod may affect some responses to cold acclimation (Stevens 1983), its effect on the development of freeze tolerance is unknown. During cold acclimation, each frog was kept in an individual container with access to water in a plastic dish, and was offered food thrice weekly until it no longer fed. Following the 8 weeks of cold acclimation, frogs (*N* = 6) remained at 5 °C for an additional 3 weeks before being sampled in early January (hereafter “cold frogs”).

A subset of frogs (*N* = 5) was cold-acclimated and kept at 5 °C for an additional 12 weeks (referred to as “long-term cold frogs”), and plasma glycerol levels were monitored in this group through repeated blood sampling. Animals were sampled for blood when their acclimation temperatures were 20, 8, and 5 °C, which resulted in frogs being sampled approximately at 4 week intervals, except for two sample points at 5 °C, which were 11 weeks apart. Blood was sampled by puncturing an axillary blood vessel, followed by collection (of approximately 30 µl) into heparinized microcapillary tubes. Blood was also obtained following terminal sampling of this group at the end of the 12 weeks at 5 °C. Following collection, blood was centrifuged (2000*g*, ~ 3 min) to isolate the plasma, and frozen in liquid N_2_.

### Freezing experiments

Cold frogs were used in freezing and thawing experiments in early January. The cold frogs from the *Acclimation experiment* served as the unfrozen controls (*N* = 5–6). Animals to be frozen were kept in individual plastic containers and were placed in an incubator set at 2 °C. The temperature of the incubator decreased 1 °C day^−1^ until it reached − 2 °C. Once the incubator reached − 2 °C, the unfrozen frogs were each quickly placed on top of moist gauze and ice was added to the individual containers to ensure crystallization. Immediately after ice was added to the containers, the incubator temperature was lowered to − 2.5 °C. Frogs were visually inspected 24 h post-inoculation for signs of internal ice formation and a group (*N* = 5) was sampled shortly thereafter (“frozen frogs”). A subset of frozen frogs (*N* = 6) was sampled after thawing for 24 h at 5 °C (“thawed frogs”).

### Tissue sampling

Frogs in the *Acclimation* and *Freezing experiments* were euthanized following approved LACUC protocols. Warm, cold, long-term cold, frozen, and thawed frogs were dissected in a 4 °C room after being weighed and their bladder fluid removed (bladder fluid was not removed in frozen frogs). Blood was collected from severed vessels, or from the heart (in frozen frogs), as described above. The liver and skeletal muscle from the thigh were excised, and samples of these organs were immediately frozen in liquid N_2_ and stored at − 80 °C before metabolite analyses were carried out. Other portions of liver, thigh skeletal muscle, and the frog carcasses, were blotted to remove excess moisture, weighed, placed in a 65 °C oven, and reweighed after drying for 5 days. Initial water concentration in the tissues and carcass, the latter serving as a measure of body water content, was determined by dividing the mass lost after drying by the mass of the remaining dried tissue. Water content was expressed in mass (g) of water per mass (g) of dry tissue so that the magnitude of change in this variable could be readily interpreted. This was preferred over reporting water content as percent of body mass, a form of quantification that may lead to erroneous interpretation of results, as the relationship between this variable and the expression of water on a mass basis is not linear, but asymptotic.

### Metabolite analyses

Plasma osmolality was measured by freezing point depression osmometry (model 3320, Advanced Instruments, Norwood, MA, USA). Liver and skeletal muscle extracts were prepared by homogenizing samples in cold 7% (w/v) perchloric acid, followed by neutralization of the aqueous portion of the homogenate with KOH. Neutralized extracts and plasma were assayed for glycerol using a colorimetric glycerol assay reagent (Sigma Aldrich, St. Louis, MO, USA) following manufacturer’s instructions. In addition, levels of glucose, urea, and lactate in plasma and in neutralized tissue homogenates were determined using spectrophotometric assay kits (Pointe Scientific, Canton, MI, USA). Extracts of liver and skeletal muscle were also assayed for glycogen using an enzymatic procedure as previously described (do Amaral et al. 2016). Deproteinized liver and skeletal muscle extracts were neutralized with KOH and incubated at 40 °C for 2 h in a 0.2 mol l^−1^ sodium acetate buffer, pH 4.8, with 1 mg ml^−1^ amyloglucosidase (Sigma Aldrich). The reaction was stopped by adding cold 7% (w/v) perchloric acid and the free glucose was determined as described above. Glycogen concentration was expressed as glucosyl units (µmol g^−1^ dry tissue) after subtraction of the initial free glucose. Liver and skeletal muscle metabolite levels were expressed per g of dry tissue.

### Cryoprotectant tissue gradients and concentrations

To determine the direction of the tissue–plasma gradient of cryoprotectants, hepatic and muscle concentrations of these metabolites (in µmol g^−1^ tissue) were converted to µmol ml^−1^ of tissue water. This expression of metabolite concentration serves as an estimate of intracellular cryoprotectant levels and allows for direct comparison of hepatic and muscle levels with plasma values (Driedzic and Short 2007; Costanzo et al. 2013). Additionally, we statistically compared intracellular levels of glycerol and glucose.

### Statistical analysis

Means ± standard error of the mean (SEM) are the descriptive summaries used for the variables measured. Comparisons between two treatments were performed using two-sample *t*-tests. Comparisons of variables among treatments and between tissues were performed using a two-way repeated-measures analysis of variance (ANOVA), where tissue was the repeated factor (as multiple tissues from the same frog were compared). If there was a significant interaction between factors (treatment and tissue), or a significant effect of one factor, data were analyzed for simple effects with a Bonferroni correction for multiple comparisons. Comparisons among three treatments were performed using a one-way ANOVA, followed by a Tukey’s HSD test. As necessary, data were transformed to fulfill the parametric tests’ assumptions. Analyses were performed using JMP (SAS, Cary, NC, USA); significance was accepted at *P* < 0.05.

### Data availability

The data that support the findings of this study are available from the corresponding author on request.

## Results

### Acclimation experiment

Frogs were acclimated to distinct thermal regimes to determine the effect of temperature on the cryoprotectant system. Warm frogs fed readily throughout their period in captivity. In contrast, cold frogs ceased feeding when acclimation temperatures reached 8 °C. All frogs survived the acclimation treatments; all were males, with the exception of one cold frog, but the data from this frog did not differ, qualitatively, from that of other frogs in the group and the animal was kept in the analyses.

Warm and cold frogs did not differ regarding body mass, body and tissue water content, hepatic and skeletal muscle glycogen levels (Table 1). Plasma osmolality, glucose, and glycerol levels did not change with cold acclimation. However, one male cold frog (*Cold 3)*, had a plasma glycerol concentration of 44.5 µmol ml^−1^ and was a statistical outlier regarding all glycerol measurements (plasma, liver, muscle), as well as plasma osmolality. This frog was excluded from analyses pertaining to glycerol and plasma osmolality. Exclusion of *Cold 3* did not change the outcome of the statistical analyses reported, with one exception noted below.

**Table 1:** Physiological variables measured in warm, cold, and long-term cold-acclimated *Dryophytes chrysoscelis*

	Warm	Cold	*P*	Long-term cold	*P*
*N*	6	5–6		5	
Body mass (g)	7.1 ± 0.6	6.3 ± 0.6	0.35	7.4 ± 0.8	0.32
Water content					
Body (g g^−1^)	3.6 ± 0.1	3.2 ± 0.2	0.18	3.6 ± 0.1	0.15
Liver (g g^−1^)	2.7 ± 0.3	2.5 ± 0.2	0.60	2.4 ± 0.1	0.73
Muscle (g g^−1^)	3.8 ± 0.2	3.7 ± 0.2	0.57	4.4 ± 0.2	0.04
Plasma osmolality (mOsm kg^−1^)	283 ± 8	301 ± 13	0.26	262 ± 12	0.07
Plasma glycerol (µmol ml^−1^)	0.1 ± 0.03	0.4 ± 0.3	0.12	0.1 ± 0.02	0.41
Plasma glucose (µmol ml^−1^)	2.0 ± 0.5	1.8 ± 1.1	0.23	1.4 ± 0.8	0.86
Plasma urea (µmol ml^−1^)	16.3 ± 2.1	36.5 ± 6.7	0.005	23.9 ± 6.8	0.16
Glycogen					
Liver (µmol g^−1^)	1784 ± 113	1581 ± 187	0.38	1882 ± 293	0.32
Muscle (µmol g^−1^)	476 ± 77	523 ± 135	0.77	198 ± 48	0.07

Data are means ± SEM. Water content is expressed on a dry tissue basis and glycogen is expressed in µmol of glucosyl units g^−1^ of dry tissue. Muscle values pertain to thigh skeletal muscle. Two-sample *t*-tests were used to compare warm and cold frogs, and cold and long-term cold-acclimated frogs

Plasma osmolality of cold frogs was 301 ± 13 mOsm kg^−1^ and plasma glycerol levels were below 2 µmol ml^−1^ in warm and cold frogs, averaging 0.4 ± 0.3 µmol ml^−1^ in the latter (Table 1). Plasma urea concentration in cold frogs was twice that of warm frogs, with values averaging 36.5 ± 6.7 µmol ml^−1^.

Long-term cold and cold frogs were indistinguishable regarding all variables except water content in skeletal muscle, which was higher in long-term cold frogs (Table 1). Repeated sampling of long-term cold frogs revealed no change (*F*_4,1_ = 88.3, *P* = 0.46) in plasma glycerol levels, which were below 2 µmol ml^−1^ in all frogs (data not shown).

**Fig. 1:**
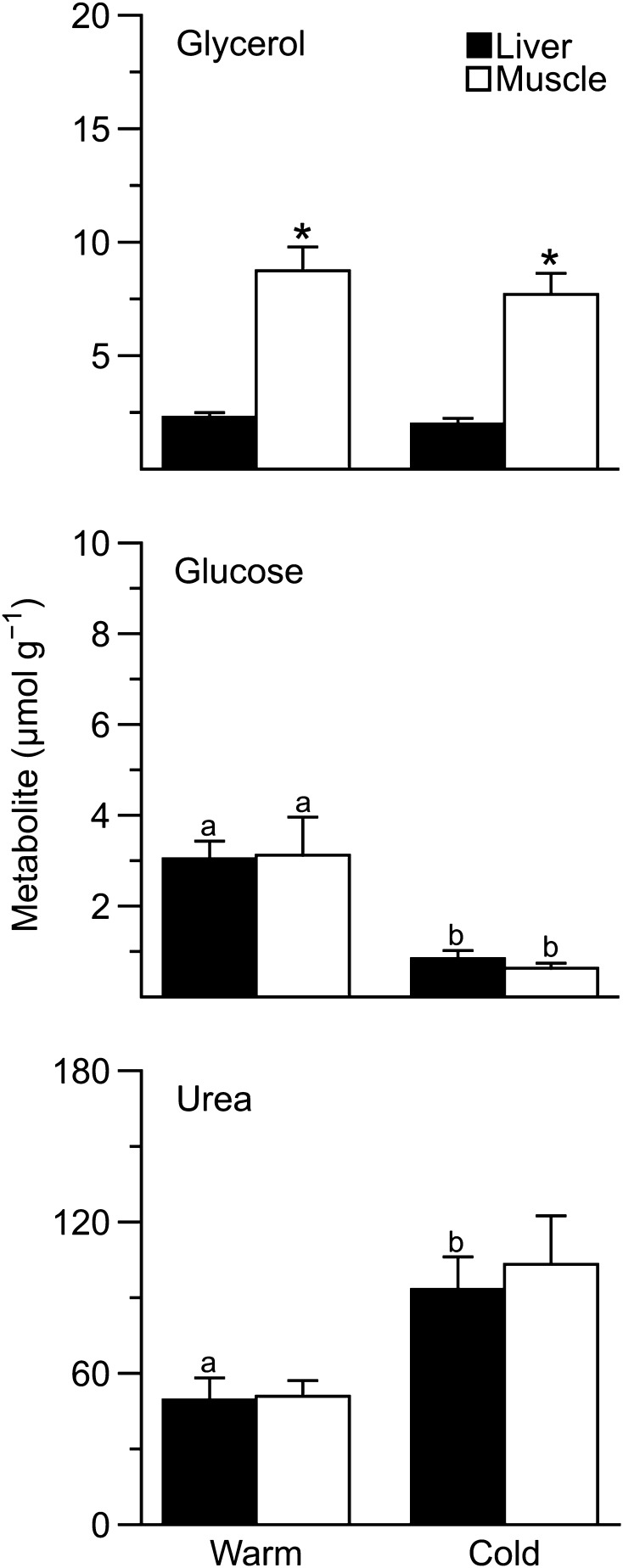
Effect of temperature acclimation on tissue glycerol, glucose, and urea levels in *Dryophytes chrysoscelis*. Liver and muscle metabolite levels in warm- and cold-acclimated frogs (mean ± SEM; *N* = 5–6) are expressed as µmol g^−1^ dry tissue. Asterisks indicate differences between liver and muscle levels (*P* < 0.05). Within a tissue, different letters indicate different concentrations between treatments (*P* < 0.05). Note the different *y*-axis scales among graphs

Tissue glycerol levels did not change (*F*_1,9_ = 1.2, *P* = 0.3) with cold acclimation, and glycerol content of cold frogs averaged 2.0 ± 0.3 and 7.7 ± 1.1 µmol g^−1^ of dry tissue in liver and muscle, respectively (Fig. 1). However, frog *Cold 3* had glycerol levels of 55.3 and 71.3 µmol g^−1^ of dry tissue in liver and skeletal muscle, respectively. Muscle glycerol levels were approximately twice those in liver (*F*_1,9_ = 161, *P* < 0.0001) in both warm and cold frogs.

Cold acclimation decreased (*F*_1,10_ = 38.3, *P* = 0.0001) hepatic and muscle glucose content, although glucose levels did not differ between tissues (Fig. 1). In contrast, levels of urea increased (*F*_1,10_ = 8.3, *P* = 0.02) with cold acclimation, in liver (*t* = – 2.9, *P* = 0.03) but not skeletal muscle (*t* = – 2.5, *P* = 0.06); nonetheless, urea levels were not distinct (*F*_1,10_ = 0.5, *P* = 0.5) between these tissues.

### Freezing experiments

All frogs subjected to freezing were males. Frogs displayed internal ice formation 24 h post-inoculation, as determined by their rigid body, stiff limbs, and presence of subcutaneous ice crystals noticeable on the ventral and dorsal surfaces. Upon dissection, visual inspection of frozen frogs revealed large pieces of ice in the coelomic cavity, sheets of ice between the skin and skeletal muscles, and ice between the muscle fibers in the hindlimbs. The cold frogs from the *Acclimation experiment* served as the unfrozen control in the freezing and thawing experiments.

Frogs that were frozen did not differ in body mass, nor body water content, from cold animals (Table 2). Hepatic water content decreased during freezing to 64% of cold frog values, but returned to baseline levels during thawing, whereas water content in skeletal muscle did not change with freezing or thawing. Freezing induced a rise in plasma osmolality, which doubled from cold to frozen frogs, followed by a modest decrease during thawing, to levels not statistically distinct from those in cold frogs. Plasma lactate concentrations increased during freezing to 15.8 ± 4.0 µmol ml^−1^, and decreased with thawing, remaining elevated above values measured in cold frogs. In frozen frogs, hepatic glycogen content decreased to about half of cold frog values, and remained low during thawing; in contrast, muscle glycogen levels did not change with freezing or thawing.

**Table 2:** Physiological variables of cold, frozen, and thawed *Dryophytes chrysoscelis*

	Cold	Frozen	Thawed	*P*
*N*	5–6	5	5–6	
Body mass (g)	6.3 ± 0.6	6.3 ± 0.5	7.0 ± 0.3	0.53
Water content				
Body (g g^−1^)	3.2 ± 0.2	3.1 ± 0.3	3.5 ± 0.2	0.50
Liver (g g^−1^)	2.5 ± 0.2^a^	1.6 ± 0.1^b^	3.0 ± 0.2^a^	0.0005
Muscle (g g^−1^)	3.7 ± 0.2	2.9 ± 0.2	3.9 ± 0.4	0.07
Plasma osmolality (mOsm kg^−1^)	301 ± 13^a^	664 ± 63^b^	429 ± 43^a^	0.0003
Plasma lactate (µmol ml^−1^)	0.8 ± 0.2^a^	15.8 ± 4.0^b^	3.5 ± 0.5^c^	< 0.0001
Glycogen				
Liver (µmol g^−1^)	1581 ± 187^a^	850 ± 127^b^	908 ± 155^b^	0.01
Muscle (µmol g^−1^)	523 ± 135	407 ± 126	344 ± 98	0.56
Glycerol				
Plasma (µmol ml^−1^)	0.4 ± 0.3^a^	149 ± 40^b^	116 ± 29^b^	< 0.0001
Liver (µmol g^−1^)	2.0 ± 0.3^a^	155 ± 27^b^	212 ± 45^b^	< 0.0001
Muscle (µmol g^−1^)	7.7 ± 1.0^a^	207 ± 51^b^	236 ± 39^b^	< 0.0001
Glucose				
Plasma (µmol ml^−1^)	1.8 ± 1.1^a^	58.8 ± 17.6^b^	18.2 ± 5.3^b^	< 0.0001
Liver (µmol g^−1^)	0.8 ± 0.2^a^	143 ± 26^b^	32.6 ± 8.9^c^	< 0.0001
Muscle (µmol g^−1^)	0.6 ± 0.1^a^	28.0 ± 7.6^b^	18.0 ± 4.0^b^	< 0.0001
Urea				
Plasma (µmol ml^−1^)	36.5 ± 6.7	53.0 ± 8.2	32.4 ± 6.8	0.17
Liver (µmol g^−1^)	93.3 ± 12.9	97.4 ± 15.5	67.5 ± 10.6	0.16
Muscle (µmol g^−1^)	103 ± 19	81.1 ± 9.8	83.6 ± 15.5	0.71

Data are means ± SEM. Water content, and liver and muscle metabolites are expressed on a dry tissue basis; glycogen is expressed in µmol of glucosyl units g^−1^ of dry tissue. Muscle values pertain to thigh skeletal muscle. Comparisons among the three treatments were performed using ANOVA followed by Tukey HSD test. Different letters indicate groups that are distinct (*P* < 0.05)

Plasma glycerol concentration increased by almost 370-fold, from 0.4 ± 0.3 to 149 ± 40 µmol ml^−1^, during freezing, and remained above baseline (*P* = 0.0009) following 24 h of thawing (Table 2). Glycerol levels in liver and muscle increased during freezing with distinct dynamics (significant interaction between tissue and treatment in repeated-measures ANOVA: *F*_2,13_ = 22.4, *P* < 0.0001). Freezing induced an increase in glycerol content, which reached 155 ± 27 and 207 ± 51 µmol g^−1^ of dry tissue in liver and muscle, respectively, and remained elevated during thawing.

Plasma glucose concentration increased from an average of 1.8 ± 1.1 µmol ml^−1^ in cold frogs, to 59 ± 18 µmol ml^−1^ in frozen frogs (Table 2), and remained above (*P* = 0.0028) that of unfrozen frogs during thawing. Hepatic and skeletal muscle glucose levels changed during freezing with distinct dynamics (significant interaction between tissue and treatment in repeated-measures ANOVA: *F*_2,14_ = 7.2, *P* = 0.007). Hepatic glucose content increased by approximately 180-fold, from 0.8 ± 0.2 to 143 ± 26 µmol g^−1^ of dry tissue after 24 h of freezing, and decreased (*P* = 0.0034) with thawing, remaining above cold frog levels (*P* < 0.0001). Glucose levels in skeletal muscle increased during freezing, reaching an average of 27.9 ± 7.6 µmol g^−1^ of dry tissue, and remained elevated during thawing (*P* < 0.0001). Plasma, liver, and skeletal muscle content of urea did not change with freezing or thawing.

### Cryoprotectant concentration gradients

We calculated cryoprotectant concentrations in liver and skeletal muscle in µmol ml^−1^ to determine the direction of the concentration gradients during warm and cold acclimation, freezing, and thawing.

**Fig. 2:**
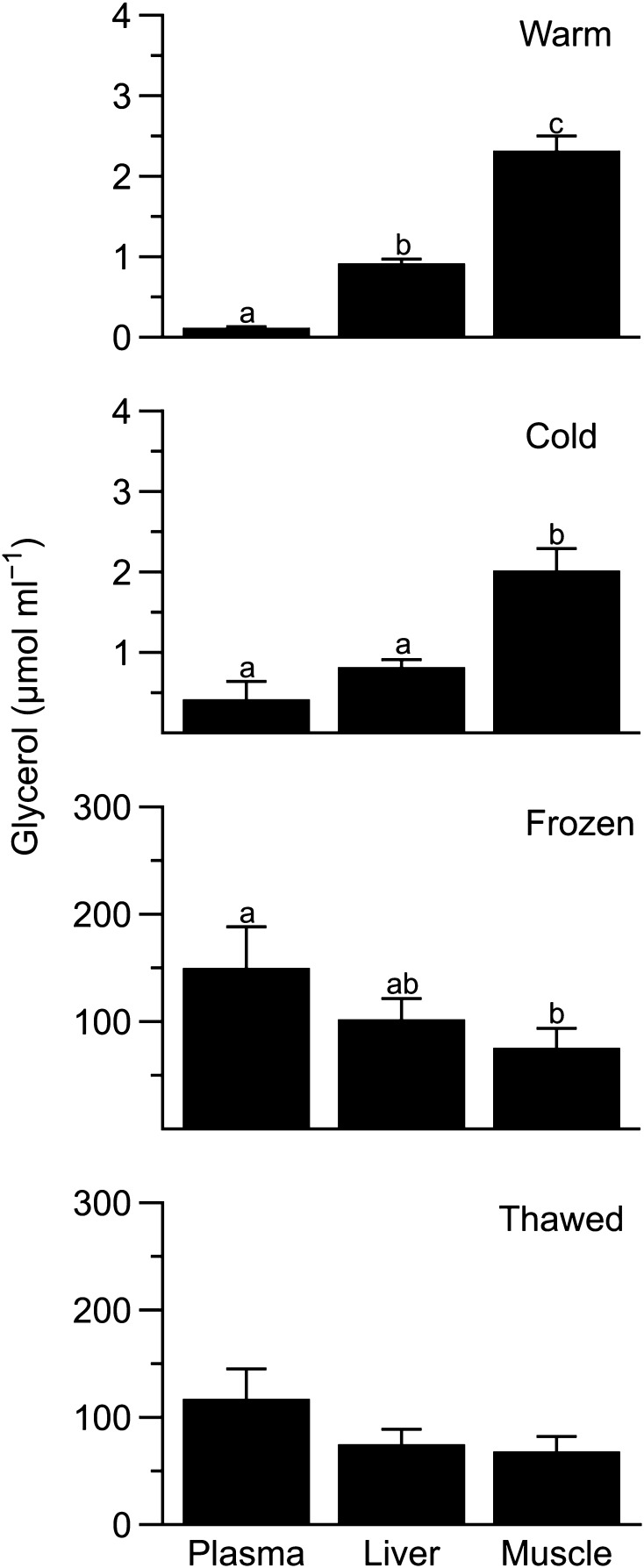
Glycerol levels in tissues of *Dryophytes chrysoscelis*. Plasma, intracellular liver, and intracellular muscle glycerol levels in cold, frozen, and thawed frogs (mean ± SEM; *N* = 5–6) expressed as µmol ml^−1^ of plasma or tissue water. Within a treatment, different letters indicate different concentrations between tissues (*P* < 0.05)

Glycerol levels varied among tissues in warm (*F*_2,4_ = 158, *P* = 0.0002) and cold frogs (*F*_2,3_ = 11.1, *P* = 0.04), being highest in muscle in both treatments (Fig. 2), except when *Cold 3* was included in the cold comparison. In frozen frogs, hepatic glycerol concentration was indistinguishable from that of plasma (*P* = 0.7), and muscle (*P* = 0.2), although plasma and muscle values differed (*P* = 0.0006). In thawed frogs glycerol concentration did not differ (*F*_2,3_ = 1.2, *P* = 0.3) among the three tissues.

**Fig. 3:**
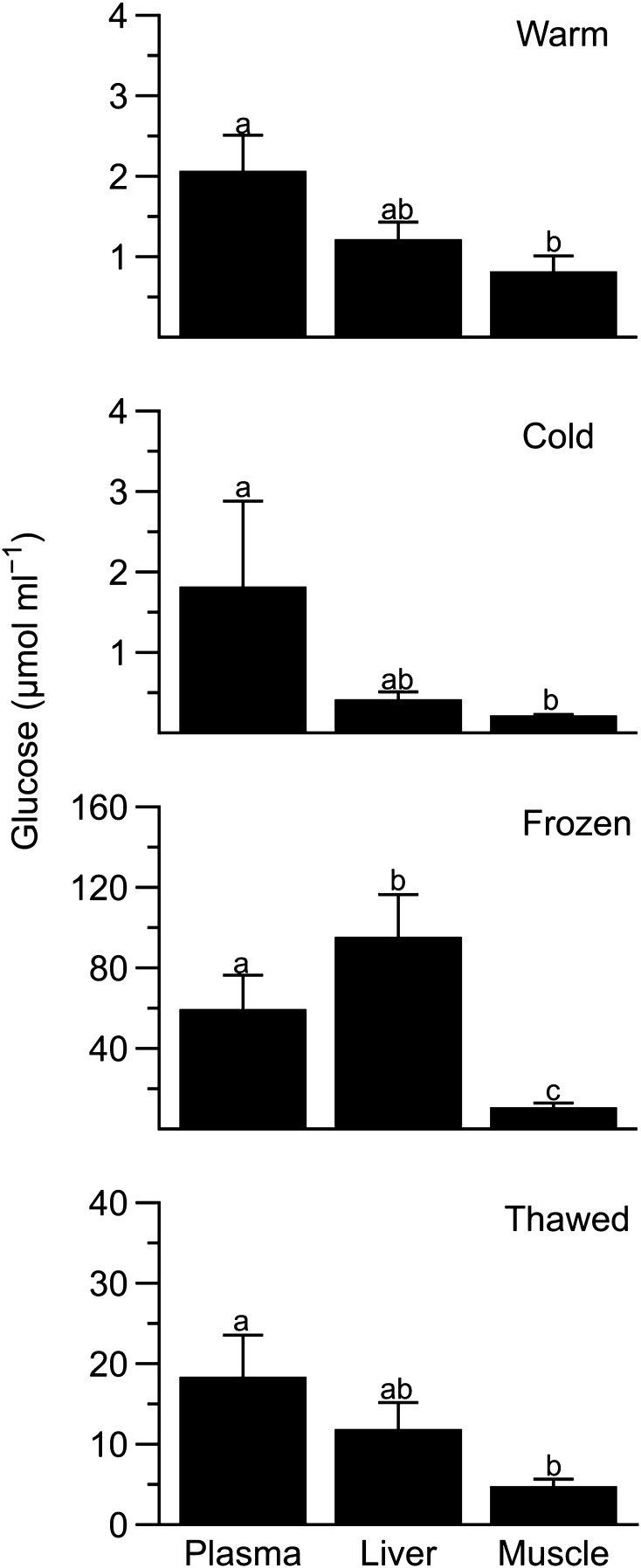
Glucose levels in tissues of *Dryophytes chrysoscelis*. Plasma, intracellular liver, and intracellular muscle glucose levels in cold, frozen, and thawed frogs (mean ± SEM; *N* = 5–6) expressed as µmol ml^−1^ of plasma or tissue water. Within a treatment, different letters indicate different concentrations between tissues (*P* < 0.05)

Although intracellular glucose concentration varied among tissues in warm (*F*_2,4_ = 14.7, *P* = 0.014) and cold frogs (*F*_2,4_ = 13.4, *P* = 0.016), plasma levels were generally higher than those in muscle (Fig. 3). Furthermore, frozen frogs had distinct glucose levels among the three tissues (*F*_2,3_ = 120, *P* = 0.0008), being lowest in muscle and approximately tenfold higher in liver. During thawing, glucose levels varied among tissues with a pattern identical to that in warm and cold animals, with hepatic and plasma levels being similar (*P* = 0.09).

The tissue concentration gradients differed between glycerol and glucose in warm (ANOVA interaction term: *F*_2,9_ = 123, *P* < 0.0001), cold (ANOVA interaction term: *F*_2,7_ = 19.2, *P* = 0.001), and frozen (ANOVA interaction term: *F*_2,7_ = 80.1, *P* < 0.0001), but not thawed (ANOVA interaction term: *F*_2,7_ = 3.4, *P* = 0.09) frogs.

**Fig. 4:**
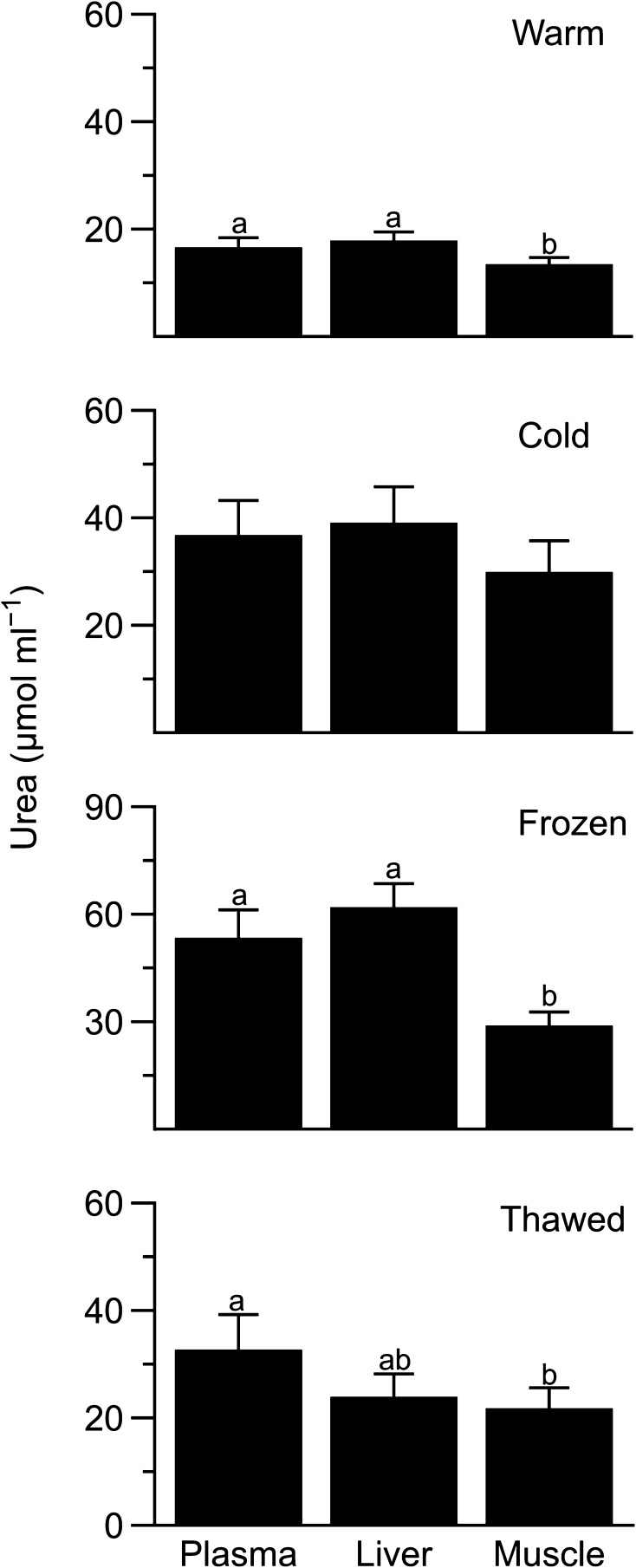
Urea levels in tissues of *Dryophytes chrysoscelis*. Plasma, intracellular liver, and intracellular muscle urea levels in cold, frozen, and thawed frogs (mean ± SEM; *N* = 5–6) expressed as µmol ml^−1^ of plasma or tissue water. Within a treatment, different letters indicate different concentrations between tissues (*P* < 0.05)

Intracellular levels of urea varied among tissues of warm (*F*_2,4_=12.7, *P* = 0.018) but not cold (*F*_2,4_ = 2.5, *P* = 0.20) frogs (Fig. 4). Plasma concentrations of urea differed from those in muscle of frozen (*F*_2,3_=44, *P* = 0.006) and thawed (*F*_2,3_ = 15.8, *P* = 0.026) animals.

### Freezing-induced cryoprotectant concentrations

Levels of freezing-mobilized glycerol and glucose were overall similar in all the tissues sampled (values shown in Figs. 2, 3). Although plasma glycerol concentration was lower than that of glucose in warm frogs (*t* = 9.1, *P* < 0.0001), plasma concentrations of these two cryoprotectants did not differ in cold (*t* = 2.2, *P* = 0.18), frozen (*t* = − 1.74, *P* = 0.33), or thawed frogs (*t* = − 2.58, *P* = 0.11). Moreover, hepatic intracellular concentrations of glycerol and glucose were similar in all treatments except in thawed frogs, in which hepatic glycerol concentration surpassed (*t* = − 3.7, *P* = 0.01) that of glucose. Intracellular muscle glycerol concentrations were higher than those of glucose in all treatments (warm: *t* = − 4.6, *P* = 0.010; cold: *t* = − 7.1, *P* = 0.0003; frozen: *t* = − 4.1, *P* = 0.01; thawed: *t* = − 6.1, *P* = 0.0003).

## Discussion

Treefrogs are unique among freeze-tolerant anurans as they not only accumulate glucose with the initiation of freezing, but may also accumulate glycerol upon exposure to low temperatures and freezing (Costanzo and Lee 2013; Storey and Storey 2017). The present study investigated the cryoprotectant system in Cope’s gray treefrog, *Dryophytes chrysoscelis*, finding unique mobilization dynamics for each cryoprotectant, and that urea likely serves as a cryoprotectant in this species.

### Effect of cold acclimation on the cryoprotectant system

Cold acclimation induces changes in gene expression, enzyme activity, and structure of lipid membranes, which prepare freeze-tolerant species to withstand not only low temperatures but also the challenges of internal ice formation (Costanzo and Lee 2013; Storey and Storey 2017). Glycerol levels were not elevated in most of cold *D. chrysoscelis*, including the long-term cold-acclimated frogs, contrasting with previous reports (e.g., Layne 1999; Zimmerman et al. 2007). While cold acclimation may induce glycerol accumulation, the variability in this response suggests that the low temperatures, reduced photophase, and aphagia experienced during this period do not guarantee glycerol accumulation (Layne and Stapleton 2008); other factors including frog energetic and osmotic status may determine cryoprotectant accumulation (Costanzo and Lee 2005).

The glycerol content in muscle tissue, albeit low, was higher than that of plasma and liver of both warm and cold frogs, as previously observed (Irwin and Lee 2003), implying glycerol production in the muscle, possibly as a result of carbohydrate or lipid catabolism (Marsh and Taigen 1987). Although certain skeletal muscles in treefrogs contain large lipid reserves (Marsh and Taigen 1987), lipid catabolism presumably has little relevance in resting metabolism of skeletal muscle in anurans (Ohira and Ohira 1988; Petersen and Gleeson 2009). Nonetheless, our results suggest muscle as an additional source for glycerol, a phenomenon that is being further investigated.

Urea levels were elevated in cold *D. chrysoscelis*. Urea reduces the equilibrium freezing point of body fluids (Clausen and Costanzo 1990), depresses metabolism (Muir et al. 2008), and preserves membrane fluidity in conditions of limited water availability (Nowacka et al. 2012), although the presence of this metabolite does not always enhance freezing survival (Higgins and Swanson 2013). In *R. sylvatica* urea is a major cryoprotectant that is accumulated during winter preparation, as a result of protein catabolism (Costanzo and Lee 2005; Costanzo et al. 2015), as is likely the case in cold-acclimated treefrogs. In addition, urea levels are possibly further enhanced by a decrease in kidney function in cold frogs (Zimmerman et al. 2007). Curiously, tissue levels of glycerol and urea were elevated in *Cold 3*, and this frog had the lowest tissue water levels of the cold group. Although dehydration of warm-acclimated animals does not induce glycerol synthesis (Zimmerman et al. 2007), mild dehydration of cold frogs may induce protein catabolism, not only promoting urea accumulation (Costanzo et al. 2015), but also supplying amino acids for hepatic glycerol synthesis (Raymond and Driedzic 1997).

Glycogen levels reported in this study were comparable to those found in previous studies, albeit slightly lower, likely due to differences in housing and acclimation procedures (Irwin and Lee 2003; Storey and Storey 1985). Following several months of cold acclimation and aphagia, glycogen stores did not change, as observed in other freeze-tolerant frogs (Dinsmore and Swanson 2008; Costanzo et al. 2013; do Amaral et al. 2016). These data suggest that basal metabolism is being supported by other substrates, possibly proteins and lipids, contrasting with previous reports of hepatic glycogen catabolism during cold acclimation and winter months (Koskela and Pasanen 1975; Schlaghecke and Blüm 1978). As hepatic glycogen reserves are important for freezing-induced mobilization of glucose (Costanzo and Lee 2013; Storey and Storey 2017), and putatively glycerol (Storey and Storey 1985; Irwin and Lee 2003), carbohydrate sparing is likely a strategy common to freeze-tolerant anurans (Costanzo et al. 2014). However, if anticipatory glycerol accumulation is the result of glycogen catabolism (Storey and Storey 1985), cryoprotectant mobilization during freezing might be compromised due to an early depletion of carbohydrates stores, a hypothesis we were unable to test as cold frogs did not accumulate glycerol.

### Cryoprotectant responses to freezing and thawing

Here we present the first demonstration of freezing-induced accumulation of glycerol in *D. chrysoscelis*, with plasma glycerol levels rising by almost 370-fold, to 149 ± 40 µmol ml^−1^, values similar to those reported for *D. versicolor* (Storey and Storey 1986; Layne and Stapleton 2008). Considering the variability in the anticipatory accumulation of glycerol, as evidenced by the current and previous studies (Layne and Stapleton 2008), some of the glycerol increases attributed to freezing may be the result glycerol synthesis during cold acclimation, although it is not possible to ascertain if this is the case in the current study as each frog was sampled only once.

Glycerol concentration in frozen frogs was similar between liver and plasma, liver and muscle, but not between muscle and plasma. This discrepancy may stem from the fact that two of the frozen frogs had plasma glycerol levels that were twice those in liver and muscle, raising the possibility of another source of glycerol, such as the fat bodies. Scarce plasma samples precluded us from determining levels of free fatty acids, which might have clarified the extent of lipid catabolism in these frogs. However, in the remaining three frozen frogs, equilibrium was reached between plasma and intracellular liver glycerol concentration, suggesting the liver is a source for glycerol in these cases; concentration of glycerol was lower in muscle, likely because circulation to this tissue was compromised early in the freeze (Rubinsky et al. 1994).

Freezing induced an increase in concentration of glucose in plasma, liver, and muscle of *D. chrysoscelis*, while hepatic glycogen concentration was reduced to 50%, and no change was observed in muscle glycogen. Levels of glucose in muscle and liver were lower than previously reported for conspecifics from Indiana (Irwin and Lee 2003). In contrast, plasma glucose levels in this study were higher than previously reported values for the sister species *D. versicolor* (Storey and Storey 1985; Layne 1999; Layne and Jones 2001; Layne and Stapleton 2008), although these result in part from freeze concentration and have limited interpretative value. The differences in tissue glucose levels between studies likely result from variation in initial glycogen stores, which determine cryoprotectant accumulation (Costanzo and Lee 1993), as well as differences in the cooling parameters, and sampling point. The pattern of glucose accumulation in *D. chrysoscelis* suggests liver glycogen as the source of glucose, as detected in other freeze-tolerant species (Storey and Storey 1985, 2004). Moreover, skeletal muscle glycogen of *D. chrysoscelis* did not respond to freezing and does not appear to contribute to the pool of cryoprotective glucose.

The dynamics of cryoprotectant mobilization differed between glucose, glycerol, and urea. Somatic freezing resulted in the increase of plasma osmolality, partly as a result of cryoprotectant production, accumulation of lactate, and of freeze concentration. Urea levels did not respond to freezing, although intracellular concentrations of this metabolite differed between liver and muscle in frozen and thawed frogs, possibly due to different degrees of tissue dehydration. In contrast, concentrations of glycerol and glucose increased in all tissues during freezing, although the glucose concentration gradient between liver and plasma was more pronounced than that of glycerol, despite similar hepatic concentrations, possibly as a result of the different export system for each metabolite. Glucose export from the liver occurs via facilitated diffusion through glucose transporters (Barnard and Youngren 1992; Rosendale et al. 2014a, b), whereas glycerol exits the liver via aquaglyceroporin 9 (Krane and Goldstein 2007; Hirota et al. 2015), the latter a presumably faster process (Cura and Carruthers 2011). Furthermore, although glucose transporter expression has not been investigated in *D. chrysoscelis*, protein levels of aquaglyceroporin 9 increase in liver of frozen *D. chrysoscelis* (Stogsdill et al. 2017). Differences in the transport kinetics, abundance, and response of these membrane proteins during freezing may contribute to the dynamics observed. The cryoprotectant concentration gradients in frozen frogs suggest both glucose and glycerol were diffusing from the plasma into muscle, although the glucose gradient was more pronounced, as plasma levels of this metabolite were fivefold those in muscle, while plasma levels of glycerol were only double of those in muscle. Of note, muscle glycerol levels of frozen frogs surpassed those of glucose by more than sevenfold, a pattern also visible in earlier studies (Storey and Storey 1985; Irwin and Lee 2003). The difference in accumulation of glucose and glycerol in muscle might not only stem from different cryoprotectant export and import systems, but may also result, to an extent, from glycerol production in muscle, as detected in warm and cold frogs. The intracellular levels of cryoprotectants were calculated without accounting for bound water: water closely associated with macromolecules and unavailable as a solvent (Storey and Storey 1988). We estimate the bound water fraction in *D. chrysoscelis* to be 13.9% (assuming an equilibrium freezing point of − 0.97 °C and a lethal ice content of 66.7% at − 5 °C), following Costanzo et al. 2013. If the bound water fraction is constant across tissues, the cryoprotectant gradients will still be present as described, although the concentrations of osmolytes will be slightly more elevated than those we report. Yet, bound water fraction may vary per tissue (Storey and Storey 1988), and because it is not known if that is the case in *D. chrysoscelis*, our ability to predict the effect of bound water on the dynamics of cryoprotectant mobilization is somewhat limited.

Glycerol likely serves as a systemic cryoprotectant in *D. chrysoscelis*, given its high concentration not only in liver and plasma, but also in muscle, relative to glucose and urea. Glycerol is a superior cryoprotectant, compared to glucose, as it not only serves as a colligative cryoprotectant, but it is also compatible with protein function, maintains redox balance, reduces free radical production during anoxia, and can preserve membrane fluidity (Yancey 2005; Nowacka et al. 2012). The accumulation of glucose in *D. chrysoscelis* may contribute to protection of core organs, such as the liver, and may serve as metabolic fuel, although its colligative effects are likely minor in muscle tissue. Indeed, urea content in frozen muscle (81.1 ± 9.8 µmol g^−1^ of dry tissue) was greater than that of glucose (27.9 ± 7.3 µmol g^−1^ of dry tissue), a pattern also observed in *R. sylvatica* (Costanzo et al. 2013).

Cryprotectant levels remained elevated in plasma of thawed frogs, rendering the animals susceptible to urinary loss of these metabolites (Layne et al. 1996), although treefrogs reduce renal filtration in cold conditions (Zimmerman et al. 2007) and may be able to reabsorb cryoprotectants from the urinary bladder, as is the case with *R. sylvatica* (Costanzo et al. 1997). The decrease in hepatic glucose concentration in thawed frogs suggests glucose is being converted back into hepatic glycogen (Costanzo and Lee 2013; Storey and Storey 2017). Elevated insulin levels during thawing possibly facilitate hepatic glycogenesis (Hemmings and Storey 1996), but likely contribute to the elevated glycerol levels observed in liver and plasma of thawed frogs, as this hormone inhibits both hepatic gluconeogenesis (Edgerton et al. 2009), and aquaglyceroporin expression in hepatic tissue (Lebeck 2014).

### Perspectives

Cold acclimation contributes to the development of freeze tolerance *in Dryophytes chrysoscelis* by promoting, under certain conditions, glycerol accumulation and, as seen in this study, the accumulation of urea, a putative cryoprotectant in this species. Variation in responses among individuals, populations, and studies suggests that in addition to cold exposure, light reduction, and aphagia, other variables may contribute to the physiological responses to cold and freezing. The differential patterns of accumulation of cryoprotectants during cold acclimation, freezing, and thawing likely represent the combined results of specific responsiveness to hormonal regulation, unique roles in cellular metabolism, and solute- and tissue-specific transport processes engaged during low-temperature exposure. The presence of such a complex cryoprotectant system probably allows *Dryophytes chrysoscelis* to augment its freeze-tolerance capacity by not only mobilizing cryoprotective glucose, but also by relying on both glycerol and urea for systemic cryoprotection.
